# Action selection performance of a reconfigurable basal ganglia inspired model with Hebbian–Bayesian Go-NoGo connectivity

**DOI:** 10.3389/fnbeh.2012.00065

**Published:** 2012-10-02

**Authors:** Pierre Berthet, Jeanette Hellgren-Kotaleski, Anders Lansner

**Affiliations:** ^1^Computational Biology, School of Computer Science and Communication, KTH Royal Institute of TechnologyStockholm, Sweden; ^2^Numerical Analysis and Computer Science, Stockholm UniversityStockholm, Sweden; ^3^Stockholm Brain InstituteStockholm, Sweden

**Keywords:** basal ganglia, behavior selection, reinforcement learning, Hebbian–Bayesian plasticity, Bayesian inference, BCPNN, direct-indirect pathway, dopamine

## Abstract

Several studies have shown a strong involvement of the basal ganglia (BG) in action selection and dopamine dependent learning. The dopaminergic signal to striatum, the input stage of the BG, has been commonly described as coding a reward prediction error (RPE), i.e., the difference between the predicted and actual reward. The RPE has been hypothesized to be critical in the modulation of the synaptic plasticity in cortico-striatal synapses in the direct and indirect pathway. We developed an abstract computational model of the BG, with a dual pathway structure functionally corresponding to the direct and indirect pathways, and compared its behavior to biological data as well as other reinforcement learning models. The computations in our model are inspired by Bayesian inference, and the synaptic plasticity changes depend on a three factor Hebbian–Bayesian learning rule based on co-activation of pre- and post-synaptic units and on the value of the RPE. The model builds on a modified Actor-Critic architecture and implements the direct (Go) and the indirect (NoGo) pathway, as well as the reward prediction (RP) system, acting in a complementary fashion. We investigated the performance of the model system when different configurations of the Go, NoGo, and RP system were utilized, e.g., using only the Go, NoGo, or RP system, or combinations of those. Learning performance was investigated in several types of learning paradigms, such as learning-relearning, successive learning, stochastic learning, reversal learning and a two-choice task. The RPE and the activity of the model during learning were similar to monkey electrophysiological and behavioral data. Our results, however, show that there is not a unique best way to configure this BG model to handle well all the learning paradigms tested. We thus suggest that an agent might dynamically configure its action selection mode, possibly depending on task characteristics and also on how much time is available.

## Introduction

**Figure 1:**
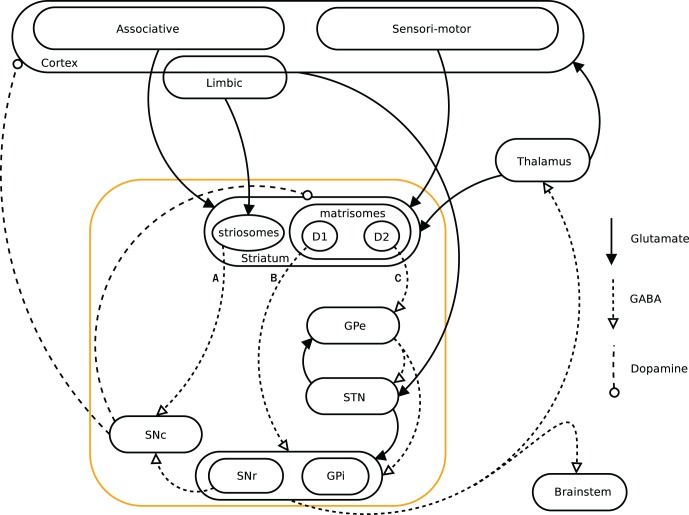
**Simplified diagram of the cortico-basal ganglia-thalamo-cortical loops.** The Basal ganglia are shown in the orange box. We present a model investigating the three pathways A (striato-SNc: symbolizes the reward prediction system), B (direct/D1: maps onto the Go pathway), and C (indirect/D2: maps onto the NoGo pathway) originating in the striatum.

When facing a situation where multiple behavioral choices are possible, the action selection process becomes critical. The ability to learn from previous experiences in order to improve further selections and their relative outcome is thus central. Basal ganglia (BG) are believed to be critically involved in action selection (Graybiel, 1995, 2005; Mink, 1996). It has been suggested that they have evolved as a centralized selection device, specialized to resolve conflicts over access to limited motor and cognitive resources (Redgrave et al., 1999). The BG structures have been conserved during evolution for more than 560 million years and are present in all vertebrates, showing a similar architecture among species (Parent and Hazrati, 1995; Grillner et al., 2005; Stephenson-Jones et al., 2011). A dual pathway architecture within BG has been described in terms of the direct- and indirect pathways. They originate from two different pools of GABAergic medium spiny neurons (MSN) expressing dopamine D1 and D2 receptors respectively (see below). Abnormalities in these pathways have been strongly linked with motor pathologies like e.g., Parkinson's and Huntington's diseases (Obeso et al., 2008; Crittenden and Graybiel, 2011). The BG receive information from different areas of the cortex, amygdala, thalamus, and dopaminergic nuclei (Parent, 1990). They are interacting with cortex and thalamus by way of several loops going through sensorimotor-, associative and limbic brain domains (Figure 1) (Alexander et al., 1986; Albin et al., 1989; McHaffie et al., 2005). Dopamine plays a key role in BG functions and is involved in the control of the different pathways (Surmeier et al., 2007), in the modulation of plasticity and learning (Reynolds and Wickens, 2002), and in coding the reward prediction error (RPE) (Montague et al., 1996; Schultz et al., 1997; Schultz and Dickinson, 2000; Daw and Doya, 2006). This RPE signal, has been used in the temporal difference (TD) learning models (Sutton and Barto, 1998) and is associated with the TD-error (Berns et al., 2001; Suri and Schultz, 2001; O'Doherty et al., 2003). Computational models have been aimed at mimicking architecture and functionality of BG, especially within the Actor-Critic and reinforcement learning frameworks (Gillies and Arbuthnott, 2000; Joel et al., 2002; Doya, 2007; Cohen and Frank, 2009; Parush et al., 2011). Various studies have furthermore shown that the central nervous system could compute and represent the world in terms of probabilities, and could perform inferences (Körding and Wolpert, 2004) similar to optimal statistical ones (Griffiths and Tenenbaum, 2006). Prior knowledge of a distribution of events would thus be combined with sensory evidence to update its representation (Friston, 2005; Yang and Shadlen, 2007). Artificial neural networks and spiking neurons have been shown to be able to code such Bayesian probabilities (Doya et al., 2007; Deneve, 2008; Buesing et al., 2011).

Here, we present and investigate a computational model of the BG based on a Bayesian inference reinforcement learning framework (Holst and Lansner, 1996). The basic idea is that the brain builds a model of the world by computing probabilities of occurrences and co-occurrences of events, storing these in the form of modified synaptic weights and neuronal excitabilities (biases). This learning framework is superimposed on a biologically well supported dual pathway architecture of the BG, which enables comparisons with biological reward learning studies and the modeling of effects of lesions and diseases of the BG. The Bayesian–Hebbian learning rule used has previously been evaluated in associative and working memory models of cortex (Sandberg et al., 2002; Johansson and Lansner, 2007; Lansner, 2009; Lundqvist et al., 2011) and here we demonstrate that it can also be used to model reinforcement learning. We here focus on the biological plausibility of the model and how the performance of its different action selection modes, i.e., how different combinations of the activation of Go and NoGo pathways and RP, perform in various reward learning tasks and compared to animal experiments.

## Materials and methods

### An abstract model of basal ganglia

Information about the current state of the world and internal state of the agent is received by the BG from the cortex and thalamus (Figure 1). BG would then provide the selection mechanism to decide on the best action to perform, given that information. It has been shown that neurons in the striatum can encode state and action value (Samejima et al., 2005; Schultz, 2007; Lau and Glimcher, 2008).

The output nuclei of the BG, internal segment of the globus pallidus (GPi) and substantia nigra pars compacta (SNr), project to the thalamus and also to the brainstem motor command centra (Hoover and Strick, 1993). The high resting activity of these nuclei keeps the target motor structures under tonic inhibition. An action can be performed when inhibition from the output nuclei of the BG is removed, i.e., when the motor command centra are disinhibited. The inhibition from the BG output nuclei can be decreased via the direct pathway, and enhanced via the indirect pathway. The MSNs associated with the direct pathway send connections mainly to SNr and GPi, while those associated with the indirect pathway project to the external part of the globus pallidus (GPe) (Gerfen et al., 1990; Parent and Hazrati, 1995). GPe in turn provides an additional inhibitory stage before projecting to SNr and GPi either directly or via the glutamatergic sub-thalamic nucleus (STN). Studies have shown that despite the fact that interactions occur between the direct and indirect pathways, activating direct pathways MSNs facilitates an action whereas activation of indirect pathway MSNs inhibits the targeted action (Gerfen et al., 1990; Kravitz et al., 2010).

**Figure 2:**
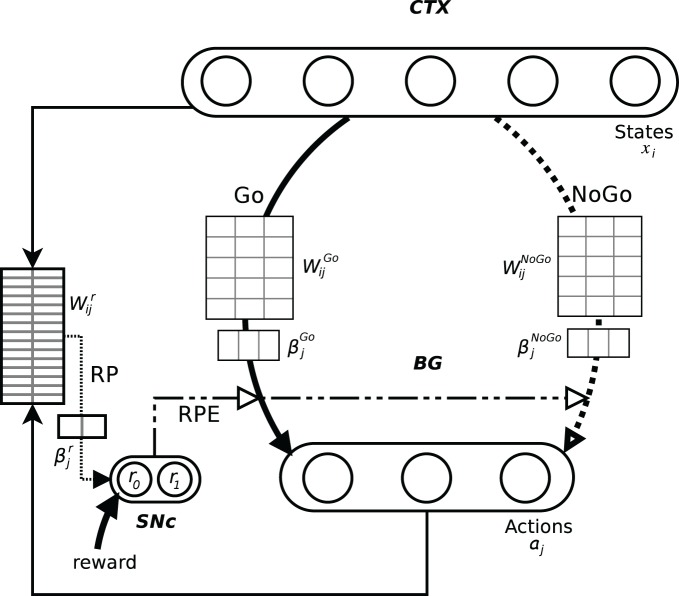
**Schematic representation of the model and its pathways.** The output layer should be seen as the whole basal ganglia. The Go and NoGo connections are all to all from the input layer, here with 5 states, to the output layer, here with 3 actions. The information about the current state and the selected action is conveyed to RP. Its weights matrix represents all the 15 possible state-action pairings. The difference between the actual reward and its predicted value is fed back to the whole system where it impacts the weights update and can also be involved in action selection (dot dashed lines).

In order to investigate how BG perform action selection, we implemented and investigated an abstract, Actor-Critic inspired, computational model, with assumed Hebbian–Bayesian plasticity in the three pathways indicated in Figure 1 (Sandberg et al., 2002). The model represents cortex and the BG as two separate populations, with units coding for states and actions in a grandmother cell-like unary representation in cortex and BG, respectively (Figure 2). Based on results from biological studies of the BG, we have implemented two pathways, one excitatory (Go) and one inhibitory (NoGo), that are considered critical for the actual selection of the actions such that the Go pathway selects which action to perform while the NoGo pathway can actively prevent non-compatible actions from being selected. A third functional pathway via the reward prediction (RP) population is configured as a feedback loop which computes the RPE, i.e., the discrepancy between the expected reward while being in a specific state and performing a selected action, and the actual reward received. In our model, this difference plays an important role in the update of the weights in both the Go, NoGo, and RP pathways. In classical Actor-Critic models, the Critic evaluates state values whereas in our model RP predicts a state-action value, similar to Q-learning and SARSA. We have investigated how the action selection performance in this model depends on the Go, NoGo, and RP capabilities under different task conditions.

We have mapped the Go pathway in our model to the direct pathway in BG (where MSNs express D1 type receptors), the NoGo to the indirect pathway (with D2 type receptors), and RP to the striato-SNc dopaminergic loop. In accordance with the biology (Aosaki et al., 1994; Calabresi et al., 1996; Schultz, 1998; Reynolds and Wickens, 2000, 2002; Kerr and Wickens, 2001; Fiorillo et al., 2003; Surmeier et al., 2007; Matsuda et al., 2009; Pawlak et al., 2010) and in a similar way to previous computational studies (Suri, 2002; Doya, 2007; Izhikevich, 2007; Hikosaka et al., 2008; Cohen and Frank, 2009), dopamine activation represents reward and acts as a modulator of the plasticity of cortico-striatal synapses, via the RPE. The directionality of the synaptic plasticity in the Go and NoGo pathway is set to be opposite for the same RPE signal, as inspired by experimental findings (Shen et al., 2008). In our model, this signal also plays an important role in the update of the weights in the RP pathway. We have here symbolized the RP system with striosomes controlling SNc, but this system could also include other areas in the brain that receive information about the state and action and which influence the dopamine release. Additional elaboration of the mapping between our model and actual neurobiology can be found in the “Discussion” section.

We have implemented a softmax action selection in order to single out a unique action from the action layer, despite the fact that several action units are likely to have non-zero values. It has been suggested to offer a good match with human exploratory behavior (Daw and Doya, 2006) and multidimensional optimization (Parush et al., 2011). This selection process could be explained by interneurons in striatum but could also result from recurrent lateral inhibition, or feed-forward competition along nuclei in the two pathways of the BG (Bolam et al., 2000; Gurney et al., 2001; Bar-Gad et al., 2003; Voorn et al., 2004; Chakravarthy et al., 2010).

### Formal model description

The Bayesian Confidence Propagation Neural Network (BCPNN; Sandberg et al., 2002) is used to select an action given the current state based on occurrence and co-occurrence of states, actions, and reward history. The network is made of abstract units with a graded output in [0 1] corresponding to local populations of on the order of 10–100 neurons, like e.g., a cortical minicolumn. A number of such units are collected in soft-winner-take-all modules analogous to cortical hypercolumns (Peters and Yilmaz, 1993). The network is trained using a Hebbian–Bayesian learning rule, which treats the units in the network as representing probabilities of stochastic events, and calculates the weights between units based on correlation between these events.

Suppose we need to decide to take an action *A* given a state characterized be the values of H input attributes, **X** = {*X*_1_, *X*_2_, … *X*_*H*_}. Analogous to a Naïve Bayes classification, we consider the state attributes independent both with and without the action *A* known. This means that the probability of the joint outcome ***X*** can be written as a product,

(1) $$P(X)=P(X_{1})P(X_{2})\ldots P(X_{H})$$

and so can the probability of ***X*** given each action *A*,

(2) $$P(X|A)=P(X_{1}|A)P(X_{2}|A)\ldots P(X_{H}|A)$$

with these assumptions and Bayes rule it is possible to write

(3) $$P(A|X)=P(A)\frac{P(X|A)}{P(X)}=P(A)\prod \frac{P(X_{i}|A)}{P(X_{i})}$$

Now, the action variable *A* and each state attribute *X*_*h*_ are assumed to be represented by a hypercolumn module and attribute values to be discrete coded, i.e., each value represented by one minicolumn unit (*a*_*j*_ and *x*_*h*,*i*_ respectively). Typically one unit is active (1) and the others silent (0) within the same hypercolumn. The $\frac{P\left(X_{i}|A\right)}{P\left(X_{i}\right)}$ factors can now be formulated as a sum of products:

(4) $$P(a_{j}|X)=P(a_{j})\prod_{h}\sum_{i\in S}\frac{P(x_{h,i}|a_{j})}{P(x_{h,i})}$$

where *S* is the indexes of active minicolumns. Taking the logarithm of this expression gives

(5) $$\log P(a_{j}|X)=\log P(a_{j})+\sum_{h}\log\sum_{i\in S}\frac{P(x_{h,i}|a_{j})}{P(x_{h,i})}$$

This can now be identified with a typical neural unit update equation for calculating the support *s*_*j*_ of a unit in *A* from the activity of the N state units with activities σ_*i*_ (1 for one unit in each hypercolumn) and the biases β_*j*_ and weights *w*_*ij*_:

(6) $$\beta_{j}=\log P(a_{j})\text{ and }w_{ij}=\log\frac{P(x_{i}|a_{j})}{P(x_{i})}$$

(7) $$s_{j}=\beta_{j}+\sum_{i\text{=1}}^{N}\sigma_{i}w_{ij}$$

In this study, we avoid the independence assumptions and instead treat the combination of all attributes as one composite attribute $\hat{X}$. We then use one minicolumn to represent each combination of attribute values, i.e., a “grandmother unit” representation. Then, the only difference is that Equation 5 becomes

(8) $$\log P(a_{j}|\hat{X})=\log P(a_{j})+\log\frac{P(x_{i}|a_{j})}{P(x_{i})}$$

where σ_*i*_ is 1 for the currently active state unit. A model with a distributed representation works identically, provided that the independence assumptions hold.

The input ***x*** and the output ***a*** of the system are binary vectors of respectively *n* and *m* elements representing *n* states and *m* actions. In these vectors, only one element is set to 1, representing the current state and the selected action, respectively. A trial, equivalent to updating the model by one time step, occurs, in summary, as follows: random activation of a unique unit in the state (cortical) layer, computation of the activation of units in the action layer (BG) and selection by the network of a unique action unit, computation of the RP based on this information, taking the action and receiving a reward value from outside of the system, and finally computation of the RPE and use of it in the update of weights and biases in the network (Equation 9).

With regard to plasticity of the network, we denote the different probabilities *P*(*x*_*i*_), *P*(*a*_*j*_), and *P*(*x*_*i*_|*a*_*j*_) in Equation 8 with variables *p*_*x*_*i*__, *p*_*a*_*j*__, and *p*_*x*_*i*___*a*_*j*__ and these are updated at each time step (*p*^*t* + Δ*t*^ = *p*^*t*^ + Δ*p*^*t* + Δ*t*^) using exponential running averages as

(9) $$\begin{array}{c} \Delta p_{x_{i}}^{t+\Delta t}=\frac{\kappa (x_{i}^{t}-p_{x_{i}}^{t})}{\tau_{p}}\Delta t\\ \Delta p_{a_{j}}^{t+\Delta t}=\frac{\kappa (a_{j}^{t}-p_{a_{j}}^{t})}{\tau_{p}}\Delta t\\ \Delta p_{x_{i}a_{j}}^{t+\Delta t}=\frac{\kappa (x_{i}^{t}a_{j}^{t}-p_{x_{i}a_{j}}^{t})}{\tau_{p}}\Delta t \end{array}$$

with τ_*p*_ the time constant and initial values *p*_*x*_*i*__ = 1/*n*, *p*_*a*_*j*__ = 1/*m* and *p*_*x*_*i*___*a*_*j*__ = 1/*nm* (1/*nm*, 1/2 and 1/2 *nm*, respectively, for RP). Each pathway has its own set of *p*-variables. These estimates are then used in Equation 6 to calculate biases and weights. The results are not very sensitive to the initialization as the updates converge relatively quickly with increasing number of trials. In our simulations, each pattern was active during one time step Δ*t* = 1, corresponding to the duration of one trial.

The three pathways, Go, NoGo, and RP, all work under the same principles. The action units basically sum the activation they get from each pathway (Equation 10) and do not implement any threshold or membrane potential.

For the selection of an action, the activations of the Go and NoGo pathways are usually combined. This can be done in different ways (see Table 1 below) but is most commonly done as

**Table 1:** **Specification of the different strategies to select an action**.

Actor	*s*_*j*_ = *s*^Go^_*j*_ − *s*^NoGo^_*j*_	Use Go and NoGo pathway (standard)
Actor Go	*s*_*j*_ = *s*^Go^_*j*_	Use only Go pathway
Actor NoGo	*s*_*j*_ = −*s*^NoGo^_*j*_	Use only NoGo pathway
RP	*s*_*j*_ = log(*r*_1_)|*j*	Given the current state, use the network to find the action that maximizes predicted reward. Here *j* indexes the action.
Actor + RP	*s*_*j*_ = *s*^Go^_*j*_ − *s*^NoGo^_*j*_ + log(*r*_1_)|*j*	Equal weight given to Actor and RP. Here *j* indexes the action.

The leftmost column states the name of the mode, the middle column shows how the argument of the Softmax function (Equation 11) is computed and the rightmost column provides some additional explanation and information.

(10) $$s_{j}=s_{j}^{\text{Go}}-s_{j}^{\text{NoGo}}$$

The activation *s*_*j*_ then represents the log-propensity to select action *a*_*j*_ given the current state ***X***. A softmax activation function (Equation 11) with gain γ then gives the probability distribution over *A* on which a random draw will pick the action that becomes the selected one. The action which has the highest activity is picked most of the time, but the softmax still allows some exploration by occasionally selecting a different action.

(11) $$P(a_{j}=1)=\frac{e^{\gamma s_{j}}}{\sum_{k}e^{\gamma s_{k}}}$$

The gain parameter γ was 5 in all simulations performed in this study as it gives some sharpness in the selection.

The RP layer activation is computed in an analogous way as that of the action layer but from *X* × *A* representing all possible state-action pairings. The output variable *R* is discrete coded with two units with activation *r*_0_ and *r*_1_, respectively, (see *W*^*r*^_*ij*_ of RP in Figure 2). A softmax function with gain = 1 is applied, but no random draw follows. After this, *r*_1_ represents the posterior probability of getting a reward based on previous experience. Given the actual reward *r* the RPE is computed as

(12) $$\text{RPE }=r-r_{1}$$

The RPE can be negative, which would correspond to a dip in dopamine release. κ represents an update signal variable (“learn-now”) which is controlled by the RPE as κ = η · |RPE|, where η is the learning rate, which was set to 0.1 in all simulations in this study. If the actual reward is exactly what the system had predicted, then RPE is 0 and from Equation 9 above it is trivial to see that the different *p* values will then not change and the bias and weights will stay the same.

Importantly, RPE has opposite effects on the updates of the *p*-variables for the Go and the NoGo pathway. If the RPE is positive (negative), the corresponding activation of the Go (NoGo) pathway are updated as described previously. However if the value passed is negative (positive), the binary vector ***a*** of the Go (NoGo) pathway is changed to its complement (Equation 13).

(13) $${\bar{a}}_{j}=\frac{\left(1-a_{j}\right)}{m-1}$$

This normalizes ***ā*** such that its components sum to 1. As an example, for a negative RPE, the main effect of this is to decrease the chance of taking the previously unsuccessful action when in the same state, and to increase it fractionally for all the other actions. The behaviors of the Go and NoGo pathways are thus asymmetrical.

### Different strategies for action selection

The action selection can be done in other ways than described above and we have investigated the performance using different strategies. These are implemented by calculating the *s*_*j*_ in different ways according to Table 1 before applying Equation 11 and selecting the action.

## Results

The performance of our action selection model inspired by the BG was evaluated with regard to the level of correct choices in a number of tasks with deterministic as well as stochastic rewards. A trial is correct when the system selects the action which has been defined beforehand as the one (see Table 2), if not the only one, leading to a delivery of the reward with the highest probability, for a given state. For the simple learning task we also measured the speed of learning, i.e., the number of trials taken to learn it. We further compared our model with data from monkey experiments by simulating a two-choice task where the reward probability was manipulated in similar ways to the experimental study by Samejima et al. (2005). Finally, we compared the development of the RPE of the model with the measured firing rate of dopaminergic neurons in monkey given the same reward delivery scheme. In the following we describe these tasks and the results achieved.

**Table 2:** **Illustration of state-action-reward mapping**.

**A**											**B**										
	***x*_1_**	***x*_2_**	***x*_3_**	***x*_4_**	***x*_5_**	***x*_6_**	***x*_7_**	***x*_8_**	***x*_9_**	***x*_10_**		***x*_1_**	***x*_2_**	***x*_3_**	***x*_4_**	***x*_5_**	***x*_6_**	***x*_7_**	***x*_8_**	***x*_9_**	***x*_10_**
*a*_1_	1					1					*a*_1_		1					1			
*a*_2_		1					1				*a*_2_			1					1		
*a*_3_			1					1			*a*_3_				1					1	
*a*_4_				1					1		*a*_4_					1					1
*a*_5_					1					1	*a*_5_	1					1				

States are shown horizontally and actions vertically, with the nonzero reward as entries in the table. Shown is an example of a state-action-reward mapping for two consecutive blocks (A and B) in the reversal and successive reward tasks with 10 states and 5 actions. Within a block, the mapping did not change. In the reversal learning task the third mapping was the same as the first panel A, whereas in the successive learning task the rewards were shifted another step to the right with wrap-around.

The same state-action-reward mapping process was used for all the tasks, except for the two-choice task. The mapping consisted of giving a reward of value 1 for exactly one correct action for each state (see Table 2 for an example). We typically had more states than actions in order to mimic the convergent structure observed in the cortico-BG system (Kincaid et al., 1998). The probability of getting a reward when a correct choice had been made, *P(r)*, could be varied between 100% and 0%. Reward was 1 with reward probability *P(r)*, and 0 otherwise. A block is the number of trials during which both the mapping and *P(r)* is kept fixed. Most of the time, all the blocks within the same simulation run have the same number of trials and the reward probability doesn't change.

### Simple learning

The size of the network was kept small in this task (10 states and 5 actions) to improve readability of the figures. One block of 200 hundreds trials was presented with *P(r)* set to 100%. Within the same block, the mapping did not change, that is, for each state, one action was rewarded with 1 while all the others gave a reward of 0 (see Table 2A). Each action thus had two states for which a positive reward was given. For each trial, the state was randomly drawn from a uniform distribution. The learning time constant τ_*p*_ was set to 32. We count as a success a trial where the correct choice has been made.

**Figure 3:**
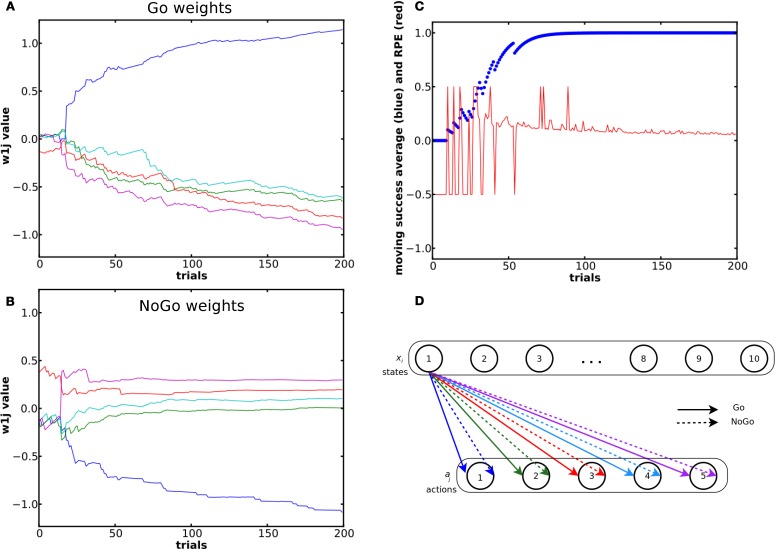
**Evolution of the weights and of the RPE during a simple learning task. (A)** and **(B)** shows dynamics of the weights from state 1 (out of 10) in the Go **(A)** and NoGo **(B)** pathways during a simple learning task. The blue curve represents the weight between state 1 and the rewarded action. **(C)** Moving average of reward over ten trials and RPE value for the same simulation. **(D)** Color coding of the different weights and the associated actions.

The results in Figure 3 come from the same single run. 176 of the 200 trials were correct. The incorrect trials (remaining 24) occurred during the initial exploratory phase. The system could learn to select the correct action for each state (see the success moving average in Figure 3C). The first choices made were purely random. When the RPE was positive, the weight between the state and the rewarded action increased in the Go pathway and decreased in the NoGo pathway and *vice versa*. During the exploratory phase, weights in the NoGo pathway showed larger amplitude variation than the weights in the Go pathway. Our interpretation is that the system first tried the red then the purple action, which led to a reward of 0, because there is a large increase in the red and purple weights in the NoGo pathway as well as a decrease in their corresponding weights in the Go pathway (see the NoGo weights dynamics in Figure 3), reducing the probability for these actions to be selected again when in the same state. When in state 1 for the third time, the blue action was tried. Due to the positive reward received, the blue Go weight increased and its NoGo weight decreased.

**Figure 4:**
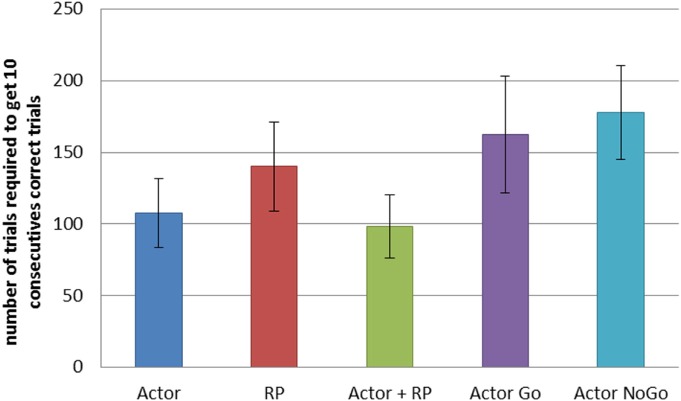
**Comparison of the learning speed of the different selection mechanisms on a simple learning task.** The reward probability was set to 100%. The standard reward mapping was used in a network of 25 states and 5 actions. Results were averaged over 200 runs. Error bars represent standard deviation. All differences are significant at *p* < 0.001, except between Actor and Actor + RP (*p* < 0.05).

We compared the different selection modes on a similar task, but with a network of 25 states and 5 actions. We recorded the number of trials required for each selection mode to reach criterion, that is to achieve 10 consecutive correct actions. We averaged the results from 200 simulations for each mode (Figure 4). A One-Way ANOVA showed a significant effect of the selection mode on the number of required trials before reaching criterion. *Post-hoc* comparisons using the Tukey HSD test indicated that all the differences between the mean number of trials required to reach criterion were significant (*p* < 0.05 for Actor ~ Actor + RP; *p* < 0.001 for the others). Actor and Actor + RP selection gave the best results, learning to select the correct action out of five possible for each of the 25 states, in on average around 100 trials, i.e., 4 trials per state. The others modes needed more trials, between 140 and 180.

### Successive learning

**Figure 5:**
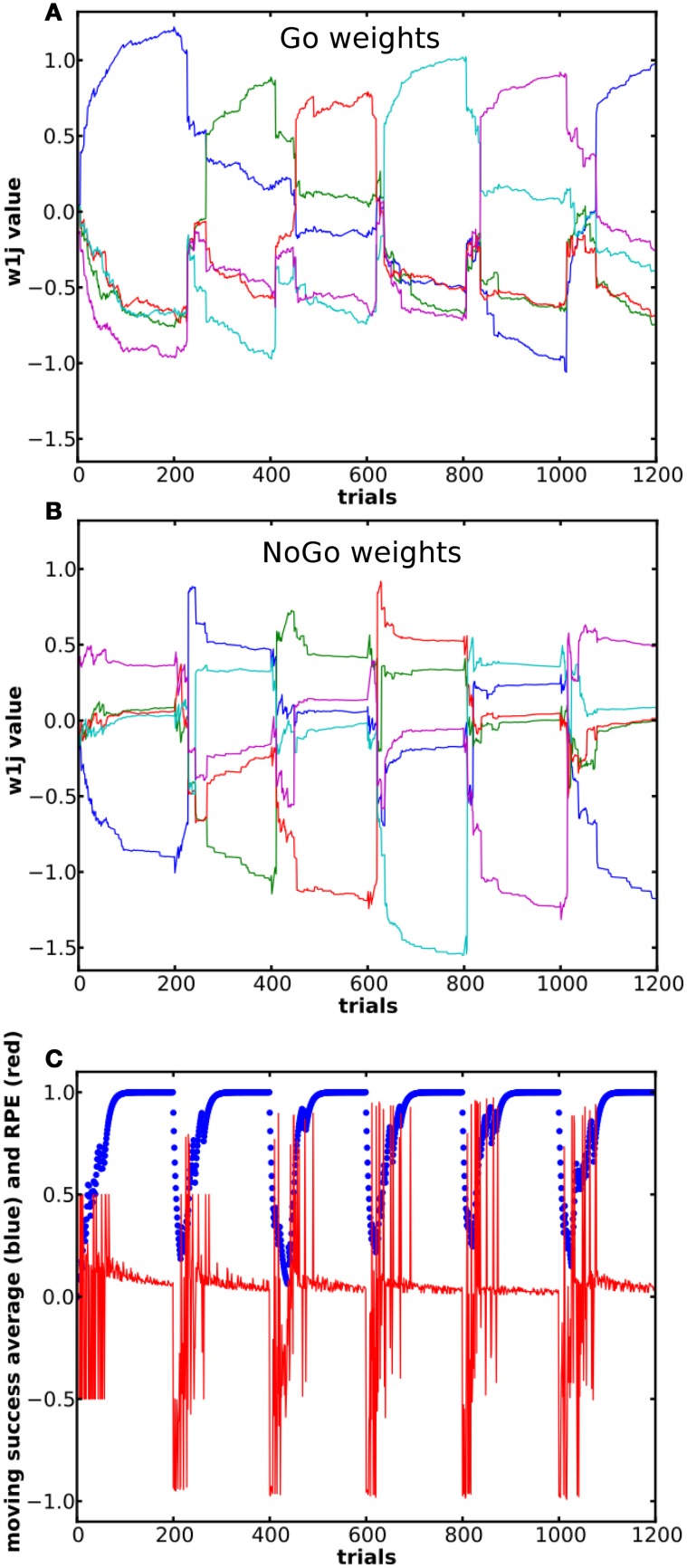
**Dynamics of the weights and of the RPE during a successive learning task.** Each block consisted of 500 trials. 6 blocks were presented, so that the reward mapping for block 1 and block 6 was the same. Panels **(A)** and **(B)** show Go and NoGo weights from state 1 to the 5 actions. Same color in the two panels represents connections to the same action. **(C)** In blue, moving average of reward over the last 10 trials. In red is shown the RPE.

In this task, the reward mapping was shifted one step every blocks of 200 trials, with wrap-around as described in Table 2, while *P(r)* was kept at 100%. The mode used was the Actor. There were 10 states and 5 actions, τ_*p*_ = 32 and the simulation consisted of 6 blocks. We measured the dynamics of the weights in the Go and NoGo pathways as well as the success rate and RPE (see Figure 5C, red curve).

The system could learn to select the appropriate action for each state, but was also able to switch its selection appropriately when the reward mapping was changed (Figure 5). This was mainly due to the fast and relatively strong increase in the NoGo weight between the current state and its previously rewarded action (see the amplitude of the change in the NoGo pathway in Figure 5B). In the Go pathway, the weight between a state and its previously appropriate action decreased as the weights from this state to the other actions increased. At some point, the weight to the previously correct action dropped sufficiently for the system to select a different one. The most dramatic updates again occurred after a change in the reward mapping. In Figure 5C, the RPE is shown and it can be seen that the RP part adjusted its prediction and gradually learned to predict an absence of reward: the RPE became less and less negative as new trials were performed after a change in the reward mapping. It can also be noted that the entropy associated with the distribution of the activation in the action layer given a specific state (results not shown here) decreases more in the Go pathway than in the NoGo pathway. This is due to the fact that the Go pathway is about promoting one action whereas in the NoGo pathway it is about suppressing all actions except one. The dynamics of the weights in the two pathways support this idea.

Furthermore, the correct mapping for the first block is learned faster (average 53.2 trials to criterion) than for successive blocks with different mappings (average 64.6 trials, Student's *t*-test, *p* < 0.001) and is probably caused by the fact that the previous mapping has to be unlearned first. This is true for all the different selection modes. Also, the size of the blocks plays a role in the performance. With 500 trials per block, the system required more trials to reach criterion in the subsequent blocks (Student's *t*-test, *p* < 0.001). This shows that even if a mapping is learned and the system always selects the correct action, significant “over-training” still occurs and the weights between the current state and the selected action are increased, making the unlearning process, required by the presentation of a new mapping, slower.

### Stochastic reward

We next compared the different action selection modes in a stochastic version of the successive learning task in which *P(r)* was modulated between 10% and 100%. We set up two versions of the task which had, respectively, 1 and 10 blocks presented for each reward probability. The system (weights and biases) was reinitialized for each change of *P(r)*. The network had 25 states and 5 actions, and each block consisted of 500 trials. Results from 20 runs for each condition were averaged and τ_*p*_ was set to 128.

**Figure 6:**
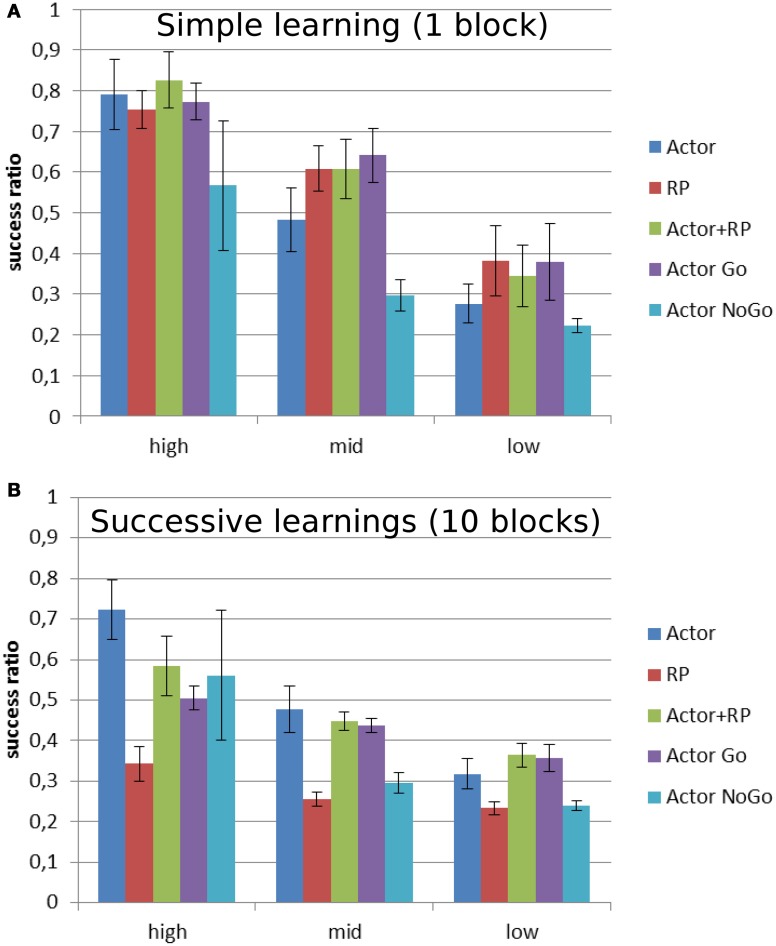
**Average success ratio of the different selection mechanisms on two versions of a stochastic reward task.** Mean success ratio and standard deviation (errors bars) are shown for three levels of reward probability: high = 100–80%, mid = 70–50%, and low = 40–10% **(A)** Stochastic simple learning: 1 block per reward probability **(B)** Stochastic successive learning: 10 blocks per reward probability. The output/actions layer consisted of 5 units, meaning that random choice would lead to a success ratio of 0.2. Success ratio is the number of correct trials over the total number of trials.

Figure 6 shows the performance as the ratio of correct over total number of trials for different levels of reward probability. The general trend is a decrease in the performance for all the modes as the reward probability decreases. In order to get a meaningful statistical analysis, the results were grouped for each of the selection modes in three groups depending on reward probability: high [100, 80], medium [70, 50] and low [40, 10]. A Two-Way ANOVA was run for each version of the task and showed a significant effect of the selection mode and reward probability, as well as their multiple interactions, on the average success ratio (number of correct trials over the number of trials). A *post-hoc* Tukey test was run to compute pairwise comparisons of the performances of the different selection modes for each level of reward probability from the two versions. When the reward level was high and only one mapping had to be learned (Figure 6A), Actor + RP performed significantly better than the others (*p* < 0.001). The biggest drop in performance is noted for Actor going from high to medium reward probability. This selection mode eventually ended as the worst performer after Actor NoGo for the low reward probabilities (*p* < 0.001).

The Actor shows a similar trend in the version with 10 blocks (Figure 6B), giving the best results for the highest level of reward probability (*p* < 0.001) and then, while it still displays the best performances for mid-level along with Actor + RP, it exhibits a decrease in its average success ratio for the lowest reward probabilities. However, in this version of the task, it is the RP mode that displays the worst performance for every level (*p* < 0.001). Only the Actor NoGo shows similar poor results for the lowest level of reward probability.

### Extinction and reacquisition

Extinction is the process by which previously established stimulus relationships are broken by the removal of reinforcers and/or biologically relevant stimuli, causing a reduction in responding. Reacquisition is the quick return of an extinguished behavior when the response and reinforcer are paired again. Studies with animals have shown that the longer the extinction is, the slower the reacquisition (Bouton, 1986; McCallum et al., 2010). Here the task was to compare the performance of the different selection modes on two different versions of an extinction task. In both cases, the simple learning, with *P(r)* of 100%, was done for 1 block of 1000 trials but the next block had a reward probability of 0% for all the choices. The number of trials in this extinction block was 0, 25, 50, 100, 500, 1000, or 2000. The third block differed such that in one version, “reacquisition,” the same mapping as in the first block was used again, whereas in the other one, “new learning,” a new mapping was used. The same type of network as in the stochastic reward tests was used, the results were averaged over 20 runs, and τ_*p*_ was set to 128.

**Figure 7:**
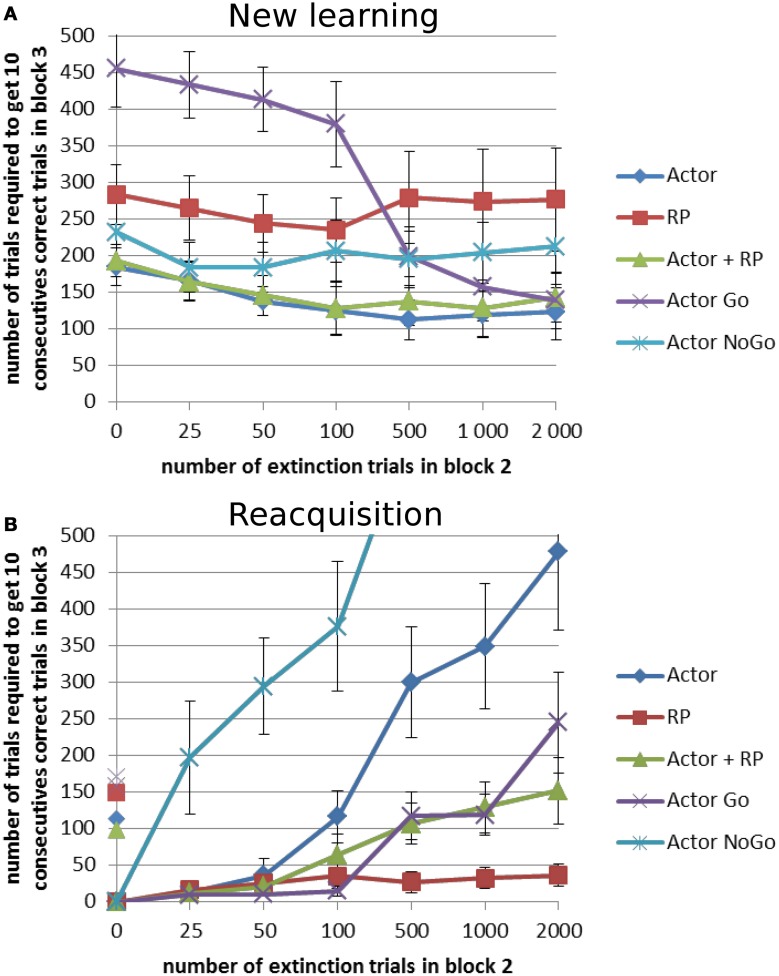
**Extinction–Reacquisition performance.** Extinction between two learning blocks results in different performances in the second block depending on the task and on the selection mechanism. During blocks 1 and 3, reward probability was 100% and it was 0% during block 2. This block could count different number of trials: 0, 25, 50, 100, 500, 1000, or 2000 trials. **(A)** New learning condition: block 3 had a different reward mapping than block 1. **(B)** Reacquisition condition: block 3 had the same reward mapping as block 1. On the y-axis, non-zero points show the number of trial needed to reach criterion for the first block for each selection mode. Error bars represent standard deviation.

The general trend in this task differs in the “new learning” and the “reacquisition” condition. The more extinction trials there are in block 2, the easier it was to learn a new mapping in block 3, especially for Actor Go (Figure 7A). When the length of block 2 was non-zero, all the results from the different selection modes were better compared to when there was no extinction trial between block 1 and 3. When the system had to reacquire the same actions as in block 1 (Figure 7B), the performance decreased with the number of extinction trials in block 2, except for the RP, quite dramatically for Actor NoGo and to a smaller extent for the Actor alone. Apart from the RP, all modes required, with variable lower extinction lengths, more trials to reach criterion in reacquisition than in the first learning in block 1.

A Two-Way ANOVA showed significant effects of the selection mode and the length of the extinction block (*p* < 0.001) for each of the two versions of the task. A *post-hoc* Tukey test was again used for pairwise comparison of the average number of trials required to reach criterion. In the new learning condition, Actor Go showed worst performance when the extinction length was the shortest (*p* < 0.001, Figure 7A). It exhibited perseverations, i.e., a tendency to keep on trying the previously rewarded actions even though they are not associated with a reward anymore, before the decrease of the weight of that action in the Go pathway enabled the system to select a different action. If the duration of the extinction was long enough to suppress the selection of that action, then the performance was quite good. This interpretation is confirmed by the opposite results that the Actor Go system got in the reacquisition paradigm. Here, it showed the best results up to an extinction length of 100 trials (*p* < 0.001, Figure 7B). Its performance gradually decreased with the number of unrewarded trials in the extinction block but stayed better than the full Actor system which was affected by the poorly performing NoGo pathway. The latter exhibited massive trouble to reacquire the correct mapping after extinction, even failing to reach criterion within the 1000 trials of the test block 3 for the longest extinction period (results not displayed in Figure 7B). This underlines the contribution of this pathway in enabling the system to switch its selection after disappointing rewards.

The RP selection displayed poor performance in the new learning condition. However, it showed the best performance in the reacquisition task (*p* < 0.001) and it was the only mode not affected by the length of the extinction in reacquisition. The effect of the length of the extinction block did not reach significance for this condition only. For this and the Actor Go mode, the performance can probably be best explained by the fact that they are quite good at learning a positively rewarded state-action mapping but worse when it comes to learn from errors. This is supported by the behavior of the Actor NoGo, which exhibited almost opposite performance to these two modes, because it learned better when the RPE was negative.

### Reversal learning

In this task, the state-action-reward mapping was changed between two different mappings, with no overlapping rewarded states (Table 2) and with 200 trials in each block. 20 blocks were presented, thus giving 10 presentations of the same mapping. The network consisted of 5 states and 15 actions, out of which only two for each state were alternatively rewarded. τ_*p*_ was set to 24.

**Figure 8:**
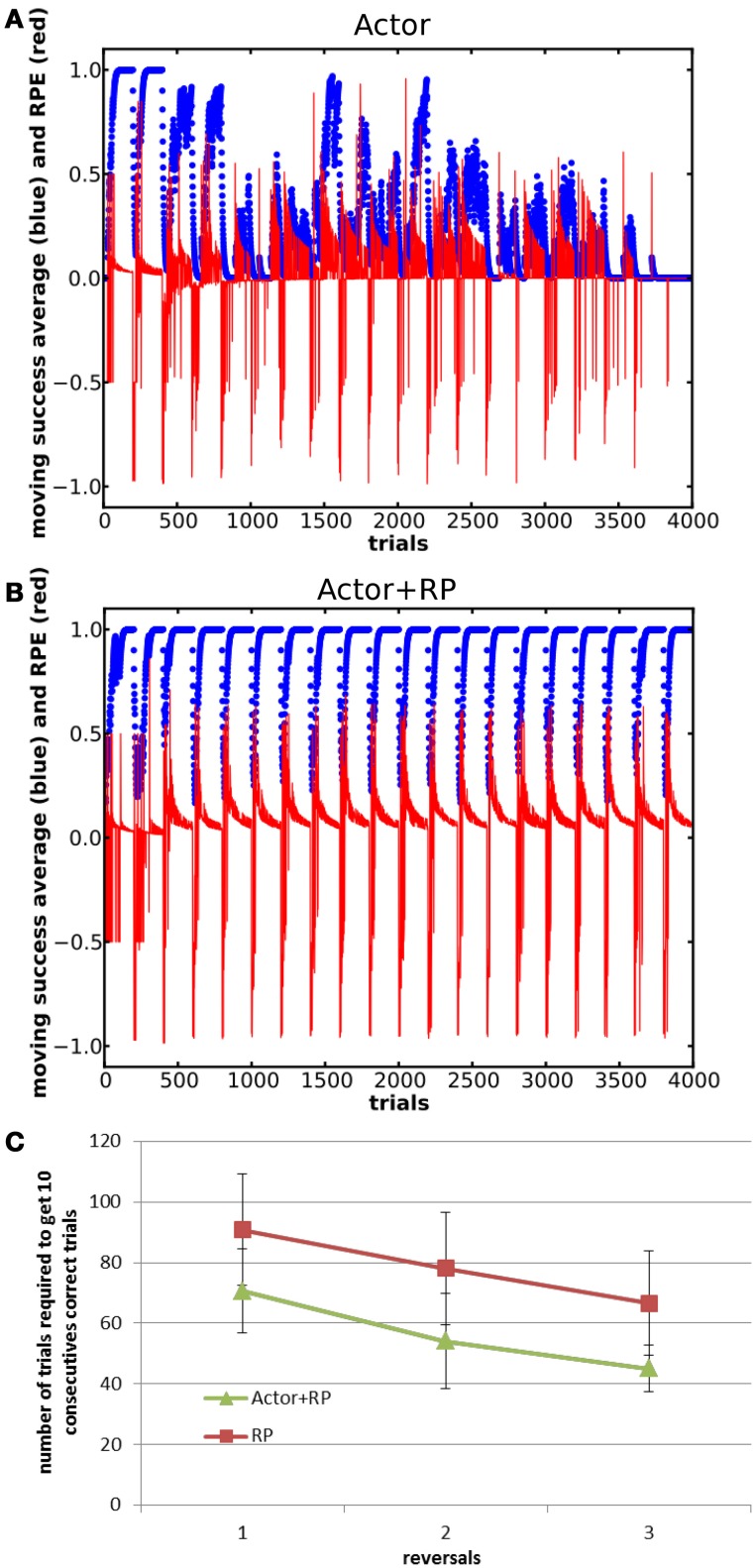
**Moving average of the success and RPE for Actor (A) and Actor+RP (B) in a reversal learning task consisting of 20 blocks of 200 trials each.** Panel **(C)** shows savings effects for Actor+RP and RP as the systems relearns the same mapping. Error bars represent standard deviation.

All the modes were able to learn the first mapping. As shown in Figure 8, the Actor showed poor performance in this task, and Actor NoGo and to a lesser extent Actor Go exhibited similar poor performances, failing to even select the correct action once for whole blocks (data not shown). Actor+RP and RP were however able to switch their selection toward the correct action along the different reversals.

In fact, all the modes that do not involve the RP in their selection mechanism failed at switching back and forth between the two mappings (results not shown here). The failure to do so lies in the dynamics of the plasticity. As a system learns that an action does not lead anymore to a reward, the associated RP of this action and the current state is decreased and the global weight, that is Go + NoGo, of this pairing is also decreased. The system can get in a position where the two actions previously rewarded have become so suppressed that all the other actions are tried instead. However, these other actions having always been associated with no reward, makes the resulting RPE approach zero. This makes the changes in the weights very small and thus keeps the system in that configuration, where the two actions that have been linked with a reward are overly suppressed. This was confirmed by a test where we increased the number of trials within a block. When large enough, the number of trials enable the system to slowly learn to suppress all the other actions that were never been associated with a reward, to the extent that the two rewarded actions became selectable again.

Moreover, we found that preventing the plasticity from becoming too low by adding a “tonic dopamine” component of about 10% to the update signal (κ) also stabilized the learning dynamics of the affected action selection modes.

We ran a Two-Way ANOVA on the number of trials needed to reach criterion for the three first odd reversals (block 1, block 3, and block 5) for the Actor + RP and the RP modes. It noted a significant effect of the mode and of the reversal number (*p* < 0.001) but not of the interaction. A *post-hoc* Tukey test supported that Actor + RP performed significantly better than RP (*p* < 0.001, see Figure 8C).

Savings are the proactive influences of prior learning on later learning even when the original behavior has been extinguished or forgotten (Kehoe and Macrae, 1997; Schmajuk, 1997). In the reversal learning context, a moderate saving effect, relatively to experimental data, was evident in the stable selection modes, resulting in a slightly faster learning of subsequent blocks compared to the first few ones (Figure 8C). We found that this was a result of traces of the previously learned state-action mapping in the weight matrix that survived past the training of the alternate mapping.

The savings seen in our model are much less prominent than what can be observed in biology and there is an absence of a clearly S-shaped learning curve. This is likely because our model represents only the final stages of learning the stimulus-response mapping and does not represent the earliest stages of the learning process, e.g., finding out and discriminating the relevant stimuli.

### Two-choice task with variable reward probability

**Figure 9:**
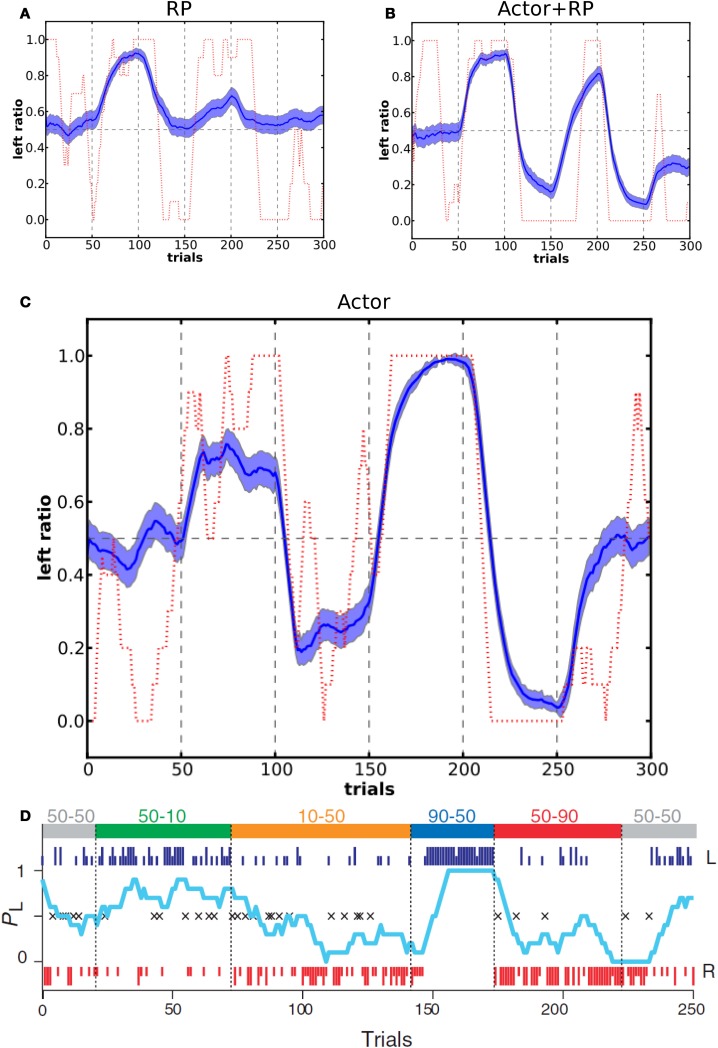
**Action selection dynamics in a two-choice task with changing reward schedule.** At the top of panel **D** are shown two values for each block, the first one is P(r|left) and the second one is P(r|right). In our simulations, each block consisted of 50 trials (blue dashed vertical lines). Panel **D** shows results reproduced from the experimental study on a monkey (Samejima et al., 2005). Panels **(A**–**C**) show the corresponding results from our model, for RP, Actor+RP and Actor respectively. Average of 100 simulations, light blue shaded area represents standard deviation and in dashed red are results from a single run. Left ratio denotes the number of left action selected trials in a window of 10 trials.

In the study by Samejima et al. (2005), the authors looked at how the probability of a monkey to perform an action, out of two possible (left and right) lever presses, was related to the probability of reward for these two actions. In order to test the choice dynamics of our model compared to the monkey we set up a network with two possible actions and followed the reward schedule given to the monkey in the experimental study. The values of the reward schedule are shown in Figure 9D. One difference is that we did not use variable block lengths but instead fixed the number of trials in a block to 50. The reason for the dynamically changing block duration in the experiment was that this prevented the monkey from learning to switch behavior relatively to the number of trials already performed, that is, to learn the number of trials within a block. In this simulation we used the Actor, Actor + RP, and RP selection modes with τ_*p*_ = 6.

Figure 9 shows the moving average ratio of left action over right action during the task, averaged over 100 runs and for a single run. The behavior of the model tended to be qualitatively similar to that of the monkey. Actor + RP selection did not show a significant difference in performance compared to Actor only. When the model was using only the RP part for the selection, it gave poor performance, that is when compared to optimal behavior, and did not show a strong analogy with the monkey behavioral responses. In accordance with results in previous tasks, the RP mode was not very efficient when mappings were often switched. It could however show good performance when *P(r)* was less than 100%. We also tested our model on a second two choice task with a different order of the blocks that was presented in their study (Samejima et al., 2005) and results were qualitatively as good as the ones showed here.

### Dopamine activation and RPE dynamics

**Figure 10:**
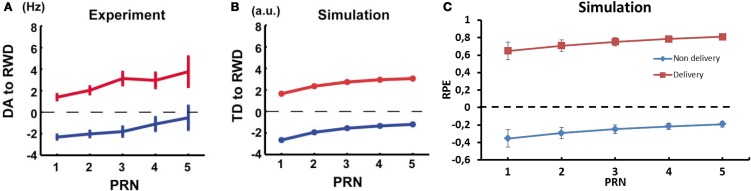
**Dynamics of RPE relatively to previous reward history and trial outcome. (A)** Experimental results of the firing rate of dopaminergic neurons from monkey and **(B)** TD error results from a conventional TD model; reward omission (blue), reward delivery (red). Reproduced from Nakahara et al. (2004) **(C)** RPE dynamic relative to PRN from our model.

Activation of dopaminergic neurons in monkey substantia nigra pars compacta (SNc) has been shown to be positively correlated with the number of preceding unrewarded trials and this could be simulated with a conventional TD model (Figures 10A,B) (Nakahara et al., 2004). In a first part, they gave a reward after a stimulus in 50% of the presentations, and used the average firing rate as baseline. They found that the firing rate of the dopaminergic neurons was linked to the recent history of reward delivery following a stimulus. The firing rate of the recorded neurons at the delivery of a reward, which followed a stimulus, increased with the number of previously unrewarded trials associated with this stimulus. Furthermore, the amplitude of the dip in firing rate noted after an unrewarded trial decreased as the number of previously unrewarded trials increased. In order to compare the results using our computational model to the results from Nakahara et al. (2004), we ran a similar test as in their study. In a first block, *P(r)* was set to 50% (baseline condition). Then, we recorded the RPE of the model both when a reward was delivered and when it was omitted. The variable was the number of unrewarded trials before the recorded trial. This post reward trial number (PRN) ranged from 1 to 5 and τ_*p*_ was set to 6.

In our model, the RPE following a rewarded trial increased for a state-action pairing as the number of previously unrewarded trials for this association increased (Figure 10C). The difference between the predicted value and the actual delivery served to update the weights in order to offer a better prediction the next time the agent was in the same situation. Similarly, the RPE decreased for the state-action pairing with each new unrewarded trial as the system adapts its RP to reflect the absence of reward. The dynamics of RPE in our model are similar to the TD-error in the standard TD-learning model used by Nakahara et al. and to the firing rate of dopaminergic neurons in the monkey (Nakahara et al., 2004).

## Discussion

The dense interactions within the BG as well as between these neural structures and other parts of the brain could provide a wide diversity of exchange and processing of information. In this work, we have focused on possible mechanisms for action selection in an abstract model of BG, investigating how information available to an agent could impact action selection based on a dual pathway probabilistic computational structure where the weights, state-action propensities, are based on learned event probabilities. Despite the abstract nature of the model, it is closer to biology than standard reinforcement learning algorithms and this could be used to help to understand diseases. In a first part, we emphasized performance assessment of the model, demonstrating that learning was possible in various tasks, where reward mapping and reward probability were probabilistic and dynamically changed. The model was able to find the correct choice and to adapt its RP such as if a discrepancy was detected, the different pathways changed their weights accordingly. It remains to compare its performances to less biologically detailed reinforcement learning models like SARSA and Q-learning. In the second part, we compared model performance with results from animal learning in a two choice tasks; the model could reproduce results from a two choice task with a dynamic reward schedule and the RPE showed the same dependence on the history of reward delivery as the activity of dopamine neurons. Future works remains to show more quantitative comparisons and details in the relation with animal learning performance.

### Relation of the model to biology

Our model was intentionally designed based on some fundamental information from biology, but it is still very abstract in nature. Some more parallels with observations from biology can, however, be identified. For instance, plasticity in the Go and NoGo pathways of the model occurred in a complementary fashion, in accordance with what had been described in biology (Shen et al., 2008). Weight updates were based on the triple factor rule: activation and co-activation of a state and an action, along with the RPE value. This mimicked the three factors observed to be critical in biology: pre- and post-synaptic activity as well as a change in baseline dopaminergic neuron firing rate (Reynolds and Wickens, 2002). The RPE in our model has as its main effect to bias the probabilities of joint activation toward state-action pairings that are positively (Go pathway) or negatively (NoGo pathway) rewarded. The dynamics of the NoGo weights showed a strong involvement in reversal and successive learning, when a previously reinforced action had to be suppressed in order to enable the system to select another one. This is similar to what has been described in biology, where D2-type receptor availability in striatum has been related to the number of trials required to switch behavior in a reversal learning condition (Groman et al., 2011).

Cortical neurons project to neurons in both the direct and indirect pathway and it is thus likely that cortical information is shared between these two pathways as in our model (Doig et al., 2010). However, sub-compartments in the striatum, striosomes (patches) and matrix, may be differentially involved in learning, in computing the RPE and selecting the action, respectively (Graybiel, 1990; Houk et al., 1995; Mengual et al., 1999). Matrisomes receive inputs from cortical sensorimotor areas and thalamus and project to downstream parts of BG. Striosomes receive information from associative and frontal cortical areas, along with amygdala inputs. Furthermore, they are projecting mostly to the SNc, which is one of the main dopaminergic nuclei in the brain and they show increased activity when a reward, or a reward predictive stimulus, is presented (Schultz et al., 2000). No direct connection was found from matrisomal neurons (Gerfen et al., 1987; Fujiyama et al., 2011) to dopaminergic neurons. For these reasons, matrisomes have been suggested to fulfil the role of the actor and striosomes have been linked with the critic part of computational Actor-Critic models.

We hypothesize that a loop including thalamus or associative- or pre-frontal cortex, sends an efference copy-like information about the selected action to the striosomes, which already have knowledge about the state to enable them to compute a prediction of the reward (Mengual et al., 1999; Haber, 2003). This view suggests that the efference copy input from motor divisions of thalamus and cortex targets striosomes, in order to contribute to the RP. This would act as an AND function of states and actions, and thus enables the RP system to know which action has been selected in the current state, in order to emit its prediction. It has been suggested that, in birds, BG circuits could receive a detailed efference copy of premotor activity (Charlesworth et al., 2012).

An important aspect of how the model is mapped to biology concerns the prominent negative part of the cortico-striatal connection matrix in the model. Most natural would be to assume this component to target feed-forward inhibition in striatum, possibly via the FS neurons (Gage et al., 2010; Planert et al., 2010). These neurons are, however, very few and it seems unlikely that they could support such a function on their own. The relative symmetry of our model opens for the possibility that the negative weights in one pathway (Go or NoGo) could in fact be positive weights in the other, a possibility that calls for further investigation.

A parameter that was varied considerably, between 6 and 128, in the tasks studied was the learning time constant τ_*p*_. This was also necessary to achieve good performance and match to experimental data. In general, small τ_*p*_ are useful for simple tasks, small number of choices and high reward schedule, as it gives large updates and thus fast learning but, when the tasks are more complex, i.e., large number of states and many possible actions and/or low reward schedule, then a long τ_*p*_ would enable the system to remember more trials and thus enable it to find the best actions among that longer memory window. A plausible possibility is the brain actually implements learning dynamics over a range of time constants while our model only represents a single one. It remains to be investigated if our model, extended with a range of plasticity time constants, would be able to solve this range of tasks without tuning of the plasticity dynamics.

### Different action selection strategies

The model we have presented here provides the possibility to combine activity in different pathways in order to perform action selection, and we investigated several different possibilities. There are two main criteria for judging which of the action selection modes studied using this model is best—accurate modeling of biology and best total performance. The most plausible and straightforward possibility is that the Actor mode is closest to what biology uses and the computations of the different actions value can be performed in parallel. The Actor Go and NoGo modes could represent lesioning or inactivation of the other pathway, rather than as intact selection mechanisms. The other possible action selection mode involves the RP, i.e., the RP, but this mode performed poorly in the stochastic successive learning task and it is also likely to be sequential in nature and take time. This is because we assume that an action would need to be imagined and its associated predicted reward stored in memory while the predicted rewards from other actions are considered, in a serial process, until all the possibilities have been compared or until time has run out. At this point the action with the highest RP could be selected. PFC might be involved in this mode of selection, since this area is more activated during tasks requiring complex selection and learning (George et al., 2011). However, when time is an issue, actions might be selected solely based on the faster parallel Actor. In fact, the most stable results over the tasks examined here were shown by the Actor + RP mode. It is unclear, however, how these different mechanisms could be combined—this may actually require a more advanced cognitive control, and it is possible that some animals lack entirely the ability to use the RP in the selection process.

The reversal learning task proved to be hard for the modes that did not use the RP for selection. These modes failed at switching back and forth between the two mappings and the failure mode was different from what is observed in animal experiments where poor performance in reversal learning is mostly ascribed to perseverative behavior (Chaves and Hodos, 1998; Judge et al., 2011). We also found, however, that adding a “tonic dopamine” component to the RPE could rescue e.g., the Actor performance. It is thus too early to state clearly even for this abstract model what constitutes the best action selection mode.

### Directions for future research

As already indicated several issues relating to the model proposed here need further investigation and validation against experimental data. The model could serve as a basis for extensions in many directions and this is necessary in order to improve performance and also the match to biological experimental data. One important neglected aspect is time—for instance reward is typically somewhat delayed relative to performance of the rewarded action. The learning rules used includes mechanisms for delayed reward so this can readily be incorporated. It could further be relevant to change the weight of the contributions from each pathway (Graybiel, 2004). Higher affinity of D2 receptors to low dopamine level compared to D1 receptors has been described (Jaber et al., 1997) which could suggest different learning rates or thresholds in the Go and the NoGo pathway.

Other important model extensions include transformation to a network model with populations of spiking neurons representing the states and actions and leaving the simple unary representations in favor of distributed representations in a spiking neural network. This as well should not meet any major obstacles and it would bring the model to a more detailed level thus making contact with experimental and modeling data at a more fine-grained biophysical level. Such an extension opens up for improved models of e.g., diseases of the BG like Parkinson's disease (PD), likely caused by a decreasing level of dopamine, resulting from the death of dopaminergic cells in SNc (Obeso et al., 2008). This could be integrated in the model in two steps: the implementation of a threshold in action selection, meaning that the activity, *s*_*j*_, would have to reach above a minimum value in order for an action to be selectable. To reproduce the low level of dopamine, the RP and the RPE should have, respectively, low and negative values. Thus, the selection modes where RP is directly involved might not reach a supra-threshold activation *s*_*j*_ in the action layer and this would thus emulate akisnesia. The Actor mode would be affected indirectly via the RPE in the update rule. This would trigger an increase in the suppressing activation from the NoGo pathway, similar to the indirect pathway over-activity remarked in Parkinsonian patients (Albin et al., 1989). It could be that if all the actions are thus depressed, they would all be below selectability threshold, eventually producing an akinesia phenomenon. Such a model might be able to shed some more light on the causes and possible treatments of this and other conditions affecting the BG.

## Conclusions

Our dual pathway model was able to rapidly find the correct state action mapping and to adapt its RP such as to solve the different action selection tasks it was evaluated on in this study. Overall, it seemed that the system combining the RP and the Go and NoGo pathways gave the best performance. It remains to be studied how such combinations could occur in biology, with respect to the type of task and to the time available for the selection for example. Furthermore, when comparing with results from animal learning experiments, the model reproduced results from a two choice task with a dynamic reward schedule and the RPE showed the same dependence on the history of reward delivery as the activity of dopamine neurons. Several extensions and much work on model validation remains for future investigations.

### Conflict of interest statement

The authors declare that the research was conducted in the absence of any commercial or financial relationships that could be construed as a potential conflict of interest.
